# Leaf trait variations associated with habitat affinity of tropical karst tree species

**DOI:** 10.1002/ece3.3611

**Published:** 2017-11-28

**Authors:** Nalaka Geekiyanage, Uromi Manage Goodale, Kunfang Cao, Kaoru Kitajima

**Affiliations:** ^1^ Division of Forest and Biomaterial Science Graduate School of Agriculture Kyoto University Kyoto Japan; ^2^ Department of Plant Science Faculty of Agriculture Rajarata University Anuradhapura Sri Lanka; ^3^ Plant Ecophysiology and Evolution Group Guangxi Key Laboratory for Forest Ecology and Conservation College of Forestry Guangxi University Nanning Guangxi China; ^4^ State Key Laboratory for Conservation and Utilization of Subtropical Agro‐Bioresources Guangxi University Nanning Guangxi China; ^5^ Smithsonian Tropical Research Institute Balboa Republic of Panama

**Keywords:** edaphic habitats, lamina thickness, leaf mass per area, photochemical reflectance index, stomatal density, stomatal pore index, trait plasticity, vein length per area

## Abstract

Karst hills, that is, jagged topography created by dissolution of limestone and other soluble rocks, are distributed extensively in tropical forest regions, including southern parts of China. They are characterized by a sharp mosaic of water and nutrient availability, from exposed hilltops with poor soil development to valleys with occasional flooding, to which trees show species‐specific distributions. Here we report the relationship of leaf functional traits to habitat preference of tropical karst trees. We described leaf traits of 19 tropical tree species in a seasonal karst rainforest in Guangxi Province, China, 12 species in situ and 13 ex situ in a non‐karst arboretum, which served as a common garden, with six species sampled in both. We examined how the measured leaf traits differed in relation to species’ habitat affinity and evaluated trait consistency between natural habitats *vs*. the arboretum. Leaf mass per area (LMA) and optical traits (light absorption and reflectance characteristics between 400 and 1,050 nm) showed significant associations with each other and habitats, with hilltop species showing high values of LMA and low values of photochemical reflectance index (PRI). For the six species sampled in both the karst forest and the arboretum, LMA, leaf dry matter content, stomatal density, and vein length per area showed inconsistent within‐species variations, whereas some traits (stomatal pore index and lamina thickness) were similar between the two sites. In conclusion, trees specialized in exposed karst hilltops with little soils are characterized by thick leaves with high tissue density indicative of conservative resources use, and this trait syndrome could potentially be sensed remotely with PRI.

## 1 INTRODUCTION

The spatial distribution patterns of tree species with respect to environmental heterogeneity are a central theme in tropical forest community ecology (Condit et al., 2000; Kitajima & Poorter, 2008; Phillips, Vargas, & Monteagudo, 2003). Many tree species exhibit strong habitat affinity to topography, elevation, and/or water and nutrient availability (Gunatilleke et al., 2006; Guo et al., 2017; Itoh et al., 2003). Such specializations occur at various spatial scales in response to edaphic habitats created by the interaction of bedrock with geomorphic factors and climate. Karst landscapes create particularly complex habitat mosaics full of fissures, conduits, sinkholes, and steep terrains, because limestone is prone to dissolution. Consequently, karst forests exhibit fine‐scale heterogeneity of hydrogeology, topography, and associated water availability, to which tree species may specialize (Bonacci, Pipan, & Culver, 2008; Guo et al., 2017; Schindler, 1982).

Identification of traits associated with edaphic habitat specialization, such as seen in tropical karst forests, may promote a mechanistic understanding of the community assembly process and ecosystem functioning. Kraft and Ackerly (2010) demonstrated how leaf mass per area (LMA) and leaf nitrogen concentrations are associated with topography in a lowland Amazonian forest in an evolutionarily convergent manner. Tree species adapted to different habitat types within a karst landscape may exhibit trait variations reflecting adaptation to differences in supply regimes of water, nutrients, and light among these habitats. As a key axis in the leaf economic spectrum (Wright et al., 2004), LMA values exhibit negative associations with inherent growth rates and soil resource availability, of which environmental heterogeneity explains around 36% of the variation (Poorter, Niinemets, Poorter, & Wright, 2009). Long leaf lifespan, which is positively associated with LMA, is adaptive in infertile soils as it reduces the nutrient turnover rates (conservative ecological strategy: Westoby, Falster, & Moles, 2002).

Optically detectable traits (hereafter optical traits) and anatomical traits may exhibit functional adaptation specific to individual environmental stress factors. Recent advances in visible to near‐infrared range (400–1,050 nm) spectroscopy allow estimation of LMA, concentrations of nitrogen and photosynthetic pigments, and water content from spectral reflectance at both leaf and canopy levels (Asner et al., 2011; Doughty, Asner, & Martin, 2010). Photochemical Reflectance Index (PRI: Gamon, Peñuelas, & Field, 1992) in particular is significant as an indicator of the degree of protection from excess radiation with xanthophyll cycle pigments. Plant adaptations to contrasting water regimes may be reflected in anatomical traits of vein and stomata, which may not be correlated with LMA or optical traits (Westoby et al., 2002). Vein length per area (VLA) of minor veins and stomatal traits are closely associated with transpiration and photosynthetic gas exchange capacity in angiosperms (Brodribb, McAdam, & Carins Murphy, 2017; Sack & Scoffoni, 2013). Vein and stomatal arrangement would indicate the supply and exchange capacity of water through leaf lamina. Functional understanding of species distributions across edaphic habitats may be advanced by assessments of these multiple leaf traits (Baltzer & Thomas, 2010).

In such trait‐based analyses, it is also important to recognize that many plant traits exhibit phenotypic plasticity (Bradshaw, 1965; Nicotra et al., 2010). Leaf traits germane to productivity and growth, such as LMA, photosynthetic capacity, and leaf lifespan, exhibit substantial plasticity in relation to heterogeneity of light and soil resources (e.g., Russo & Kitajima, 2016). It is extremely difficult, if not impossible, to separate such effects of phenotypic plasticity from inherent trait differences among species by sampling plants only in their natural habitats. Common garden experiments are useful in teasing apart whether an apparent association of certain species traits with their natural habitats is due to habitat filtering of species or plastic responses (Cordell, Goldstein, Mueller‐Dombois, Webb, & Vitousek, 1998). For tree species, an arboretum where trees were grown on a common soil and receive adequate supply of water and nutrients may serve as a common garden in which leaf traits may be evaluated under a standardized favorable condition.

Karst forests occupy 7%–15% of the global terrestrial landmass (Fu et al., 2012; Hartmann et al. 2014). In an extensive karst forest zone in southwestern China, ranging from Yunnan to Guangxi, recent studies report affinities of tree species to various topographic positions (Guo et al., 2017; Zhang, Hu, Zhu, Luo, & Ni, 2010). Steep topography and percolation of water through the bedrock accompanied by nutrient leaching and soil erosion creates a gradient of water and nutrient availability from mountain hilltops to foothills, leading to drier and nutrient‐poor conditions in hilltops and mid‐slopes compared to foothills. The karst valleys are not necessarily resource‐rich habitats due to prolonged inundation and soil anoxia in the rainy season (Bonacci et al., 2008; Guo et al., 2017; Schindler, 1982). Among the four topographical habitats we discussed above, foothills perhaps represent the most favorable in terms of water and nutrient availabilities for plant growth (Guo et al., 2015; Huang et al., 2014). However, potential associations of leaf traits in karst tree species to their habitat affinities are yet to be characterized.

Here, we report a comparative study of leaf traits among karst tree species growing in their natural habitats and in an arboretum in Guangxi Province, China. We selected 19 karst tree species known to show habitat affinity to four topographic positions (i.e., hilltop, mid‐slope, foothill, and valley). Twelve were common species in Nonggang National Nature Reserve (hereafter the karst forest), and 13 were growing in an arboretum in Nanning, Guangxi. Of these, only six species affiliated to valley and foothill habitats were sampled at both sites. We measured 13 leaf traits to test the following hypotheses: (1) species affiliated to resource‐poor hilltop and mid‐slope habitats express a suite of traits indicative of a more conservative leaf strategy such as high LMA, compared to those in more favorable foothill habitats, (2) values of a given trait of a species may differ between its natural habitat and the arboretum, but species means should show positive interspecific correlations between the two study sites. Inclusion of optically detectable traits in multivariate trait analysis in this study allowed us to explore a potential for spectral detection of suites of leaf functional traits indicative of ecological strategies and habitat affiliation.

## 2 MATERIALS AND METHODS

### 2.1 Study sites and species

**Figure 1:**
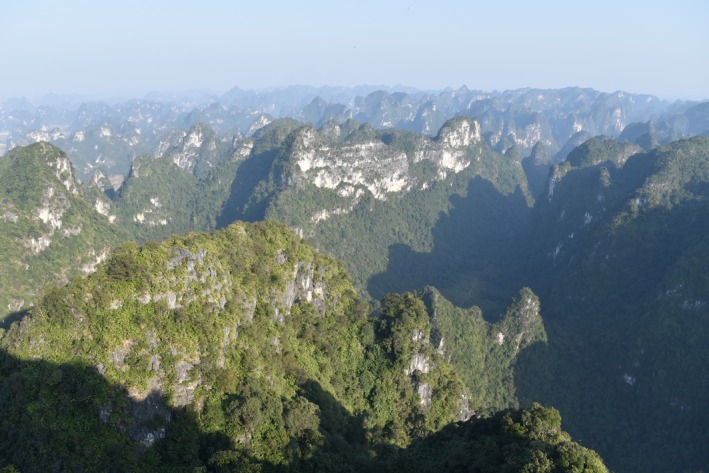
The extensive karst landscape of Nonggang National Nature Reserve, Guangxi Province, South China, seen from a hilltop. Photograph credit: N. Geekiyanage

We conducted this study in two sites in Guangxi Province in south China. The first was a seasonal karst rainforest (Figure 1, Nonggang National Nature Reserve, 22°28′ N 106°57′ E, 10,180 ha in size) encompassing a steep topographic gradient (130–607 m above mean sea level; asl). The soil volumetric water content decreases with increasing elevation in the karst forest (Fig. S1). The second was an arboretum on non‐karst soils (Qing Xiu Shan, 22°46′ N 108°33′ E, 1,354 ha in size, 80‐289 m asl). The mean annual rainfall in this region ranges from 1,200 to 1,500 mm, of which 76% falls from April to October (Wang et al., 2014). The monthly temperature means are in the range of 14–28°C (detailed climatological and other site information are in Table S1).

We selected 19 species mainly based on the information on tree dominance and habitat association available from the 15‐ha plot, which belonged to the forest dynamics network coordinated by the Center for Tropical Forest Science (Guo et al., 2015; Huang et al., 2014). Of these 19 species, we could sample 13 in the arboretum, 12 in the karst forest, and six in both sites (Table 1). These six species were affiliated to valley and foothill habitats. Trees in the arboretum had been planted approximately 30 years earlier (except for *Saurauia tristyla*, see Table 1) and watered regularly during the dry season, whereas those sampled from the karst forest had been growing in their typical habitats. We sampled three to six species in each habitat affinity class (valley, foothill, mid‐slope, and hilltop: see Table 1 for species codes, their habitat affinity, and taxonomy). Species classified to the affinity class hilltop had not been planted in the arboretum and could be sampled only in the karst forest. The set of mid‐slope species sampled in the karst forest was different from the mid‐slope species sampled in the arboretum (Table 1).

**Table 1:** Habitat affinities and taxonomy of 19 species studied in situ in Nonggang National Nature Reserve (karst forest) and ex situ in Qing Xiu Shan (arboretum), Nanning, South China. Species names follow Flora of China available online at www.efloras.org and consult the same for authority of species. All species sampled in the karst forest were grown in their natural habitats. Habitat affiliation of these (*) species were determined by the abundance of individuals in each site in consultation with local botanists

Species	Family	Habitat affinity	Species code	Sites
*Cephalomappa sinensis*	Euphorbiaceae	Valley	CESI	Arboretum
*Sterculia monosperma*	Sterculiaceae	Valley	STMO	Both
*Saraca dives*	Fabaceae	Valley	SADI	Both
*Ficus hispida*	Moraceae	Valley	FIHI	Both
*Saurauia tristyla*	Actinidiaceae	Valley	SUTH	Arboretum
*Diplodiscus trichospermus*	Tiliaceae	Foothill	DITR	Both
*Vitex kwangsiensis*	Verbenaceae	Foothill	VIKW	Both
*Camellia* sp.	Theaceae	Foothill*	CAMI	Karst forest
*Erythrina stricta*	Fabaceae	Foothill	ERST	Both
*Ficus simplicissima*	Moraceae	Foothill*	FISI	Arboretum
*Antidesma japonicum*	Euphorbiaceae	Mid‐slope	ANJA	Arboretum
*Cleistanthus sumatranus*	Euphorbiaceae	Mid‐slope	CLSU	Karst forest
*Drypetes perreticulata*	Euphorbiaceae	Mid‐slope	DRPE	Karst forest
*Excentrodendron tonkinense*	Tiliaceae	Mid‐slope	EXTO	Arboretum
*Ficus microcarpa*	Moraceae	Mid‐slope	FIMI	Arboretum
*Oroxylum indicum*	Bignoniaceae	Mid‐slope	ORIN	Arboretum
*Ligustrum* sp.	Oleaceae	Hilltop*	LIGU	Karst forest
*Viburunum propinquum*	Adoxaceae	Hilltop*	VIPR	Karst forest
*Sinsosideroxylon pedunculatum* var. *pubifolium*	Sapotaceae	Hilltop	SIPE	Karst forest

### 2.2 Sample collection

From November 2015 to January 2016, we harvested three small branches containing 5–25 sun leaves from a minimum of three mature individuals per species using a seven‐meter‐long telescopic pruner. *S. tristyla*, sampled only in the arboretum, was an exception, as it was still at the sapling stage (<5 cm dbh). All measurements were taken from mature, fully expanded, and healthy leaves following the trait measurement protocol of Pérez‐Harguindeguy et al. (2013). To maintain the water status of leaves, cut ends of branches were immediately wrapped in water‐soaked tissues and sealed in airtight polythene bags. Samples were transported from the arboretum and the karst forest in a dark cool box to the Ecophysiology and Evolution Laboratory of the Guangxi University and to the Nonggang Field Station for leaf trait measurements (Table 2).

**Table 2:** Leaf traits measured in this study, their abbreviations, units of measurements for 19 species sampled in the karst forest (in situ), and arboretum (ex situ). The last two columns are correlations between two sampling sites with bold letters indicating statistical significance (p<.05) and each data point being a species sampled at both (n = 6)

Trait and abbreviation	Units	Pearson correlation (*r*)	*p* value for *r*
Leaf area (LA)	cm^2^	.77	.073
Leaf mass per unit area (LMA)	g/m^2^	.57	.233
Lamina thickness (LT)	μm	**.98**	**<.001**
Leaf dry matter content (LDMC)	mg/g	.66	.148
Vein length per unit area (VLA)	mm/mm^2^	.43	.574
Stomatal pore index (SPI)^a^	Unitless	**.97**	**.006**
Stomatal density (SD)	#/mm^2^	.69	.193
Guard cell length (GCL)	μm	**.95**	**.011**
Chlorophyll content (Chl_SPAD_)^b^	μg/cm^2^	.51	.304
Modified chlorophyll absorption ratio index (MCARI)	Unitless	−.67	.218
Normalized difference vegetation index (NDVI)	Unitless	−.30	.625
Photochemical reflectance index (PRI)	Unitless	−.78	.118
Water band index (WBI)	Unitless	**−.94**	**.018**

a SPI calculated as SD × GCL^2^.

b Chl_SPAD_ estimated from SPAD meter. See *Materials and Methods* for details.

### 2.3 Leaf trait measurements

For six leaves per species, we measured lamina thickness (LT) with a digital micrometer (Mitutoyo Corporation, Kanagawa, Japan), fresh mass with a digital balance and leaf area (LA) with a LI 3050C leaf area meter (Li‐COR Inc., Lincoln, Nebraska, USA). After drying to a constant mass (65°C for 72 hr), we calculated leaf dry matter content (LDMC) as dry mass divided by fresh mass and LMA as leaf dry mass divided by leaf area.

Using another set of leaves, we applied clear nail polish at six to eight locations on two leaves per tree to imprint abaxial leaf surfaces for stomatal trait measurements (Lawson, James, & Weyers, 1998). None of our study species had adaxial stomata. Micro‐photographs of these imprints were taken with a digital camera mounted to a light microscope (Leica Microsystems, Wetzler, Germany), and stomatal density (SD), guard cell length (GCL), and stomatal pore index (SPI = SD × GCL^2^; stomatal pore area per lamina area) were measured. We were unable to measure stomatal traits of three species (*Ficus simplicissima, Ficus hispida* and *S. tristyla*) as clear imprints could not be obtained due to trichomes.

Additional leaf samples were preserved in formalin, acetic acid, and alcohol fixative to measure VLA (Sack & Scoffoni, 2013). Three leaf pieces per tree were chemically cleared and stained before they were micro‐photographed at high resolution (2,048 × 1,536 pixels). Micro‐photographs were analyzed with ImageJ v.1.48 (https://imagej.nih.gov/ij/). Vein traits of *Camellia* sp. and *Erythrina stricta* were not measured as samples became damaged during chemical clearing.

Prior to measuring LA, LT, LMA, and LDMC, we made nondestructive optical measurements on the same leaves. We used a SPAD meter (Minolta, Osaka, Japan) to optically estimate the chlorophyll contents per unit leaf area. Following a model by Coste et al. (2010), SPAD readings were converted to chlorophyll content (Chl_SPAD_, Appendix S1). A CI–710 leaf spectrometer (CID Bioscience, Camas, Washington USA) was used to measure light reflectance, transmission, and absorption spectra for visible (400–700 nm) to near infrared (700–950 nm) wavelengths at 0.21 nm optical resolution. From these spectral data, we calculated Photochemical Reflectance Index (PRI: Gamon et al., 1992), Modified Chlorophyll Absorption Ratio Index (MCARI: Daughtry, Walthall, & Kim, 2000), Normalized Difference Vegetation Index (NDVI: Rouse, Haas, Schell, & Deering, 1974), and Water Band Index (WBI: Peñuelas, Filella, Biel, & Serrano, 1993) (supplementary information Appendix S1 for further details).

### 2.4 Statistical analysis

All analyses were performed using R version 3.1.3 (R Core Team 2015). Some of the trait values did not follow the assumptions of normality (Shapiro–Wilk test); thus, they were scaled to zero mean and unit variance before multivariate analysis. To visualize traits' differentiation and their association with habitat affinities in the karst forest, we conducted a principal component analysis (PCA) using tree‐mean trait values (*vegan* package). We used the broken‐stick criterion to identify the main principal components responsible for trait variation (Legendre & Legendre, 2012, *BiodriversityR* package). We extracted species scores for principal axes and tested whether they significantly differed among four habitat affinity classes in linear mixed models (*lme4* package). For the separate 12 species dataset from the sampling at the karst forest and 13 species dataset from the sampling at the arboretum, a linear mixed‐effect model was fitted for each trait to test the effect of habitat affinity class (fixed effect with three levels in the arboretum and four levels in the karst forest) and species (random effect). All models were fitted with leaf‐mean trait values, and inferences were made based on chi‐square statistics (χ^2^) at .05 significant level (*lme4* package). To test the significance among habitat affinity classes, *post hoc* multiple comparisons were computed with Tukey's method at adjusted .05 significance level (*multcomp* package). To assess whether traits measured in the arboretum were correlated with their corresponding measurements in natural habitats and to test the correlations among traits measured in the karst forest, Pearson's correlation coefficients (*r*) were calculated. We used species‐mean trait values (*n* = 6) for site comparisons and tree‐mean trait values for examining bivariate trait correlations in the karst forest.

## 3 RESULTS

### 3.1 Syndromes of leaf traits across karst hill habitats

**Figure 2:**
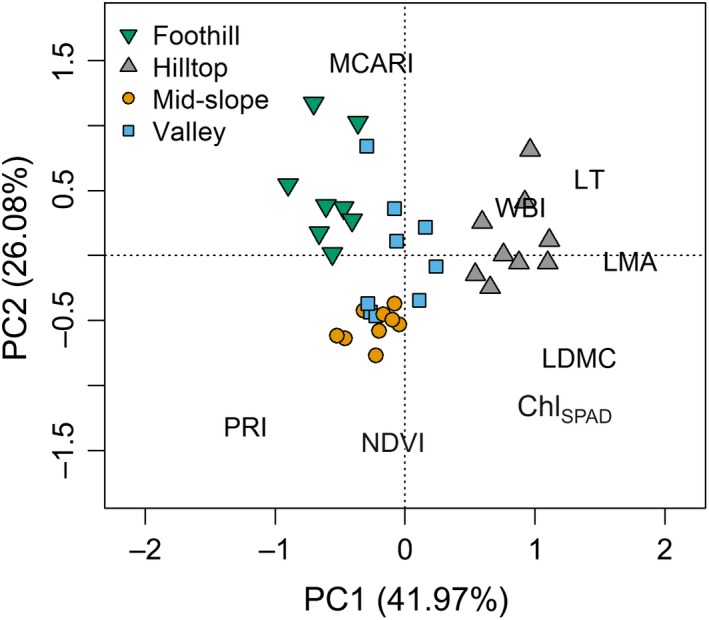
Principal component analysis (PCA) of eight leaf traits measured with 36 individuals of 12 tree species naturally growing in four karst habitats (valley, foothill, mid‐slope, and hilltop). Positions of the trait name abbreviations indicate factor loadings (further explained in Tables 2 and 3). Percentage of total variance explained by each PC axis is shown along the axis label

The eight leaf traits of 12 tree species measured in their natural habitats exhibited multivariate trait differentiation, which was greater among habitats than within‐habitats (Figure 2 showing the result of PCA). The first principal component (PC1) explained 42.0% of the variance, which was largely contributed by LMA and leaf optical traits, separating hilltop species with high LMA, LT, and LDMC from the rest (Table 3). Foothill species were clustered at the negative end of PC1, but they were not clearly segregated from mid‐slope and valley species in the middle (Figure 2). These habitat contrasts were further confirmed in a linear mixed model that tested the effect of habitat affinity on species scores for PC1 (χ^2^ = 31.65, *p* < .001). The second component (PC2) was associated with optical traits, explaining 26.1% of the variance (Figure 2, Table 3), such that greater greenness (NDVI, Chl_SPAD_) meant lower PC2 scores.

**Table 3:** Loadings of first two principal components and eight leaf traits in 12 karst tree species representing four habitat affinity classes in the karst forest. The PCA was conducted with tree level means. Trait abbreviations are given in Table 2

Trait	PC1 (41.97%)	PC2 (26.08%)
LMA	0.97	−0.02
MCARI	−0.14	0.74
NDVI	−0.05	−0.71
WBI	0.45	0.19
LT	0.80	0.29
LDMC	0.75	−0.40
PRI	−0.69	−0.65
Chl_SPAD_	0.69	−0.58

**Figure 3:**
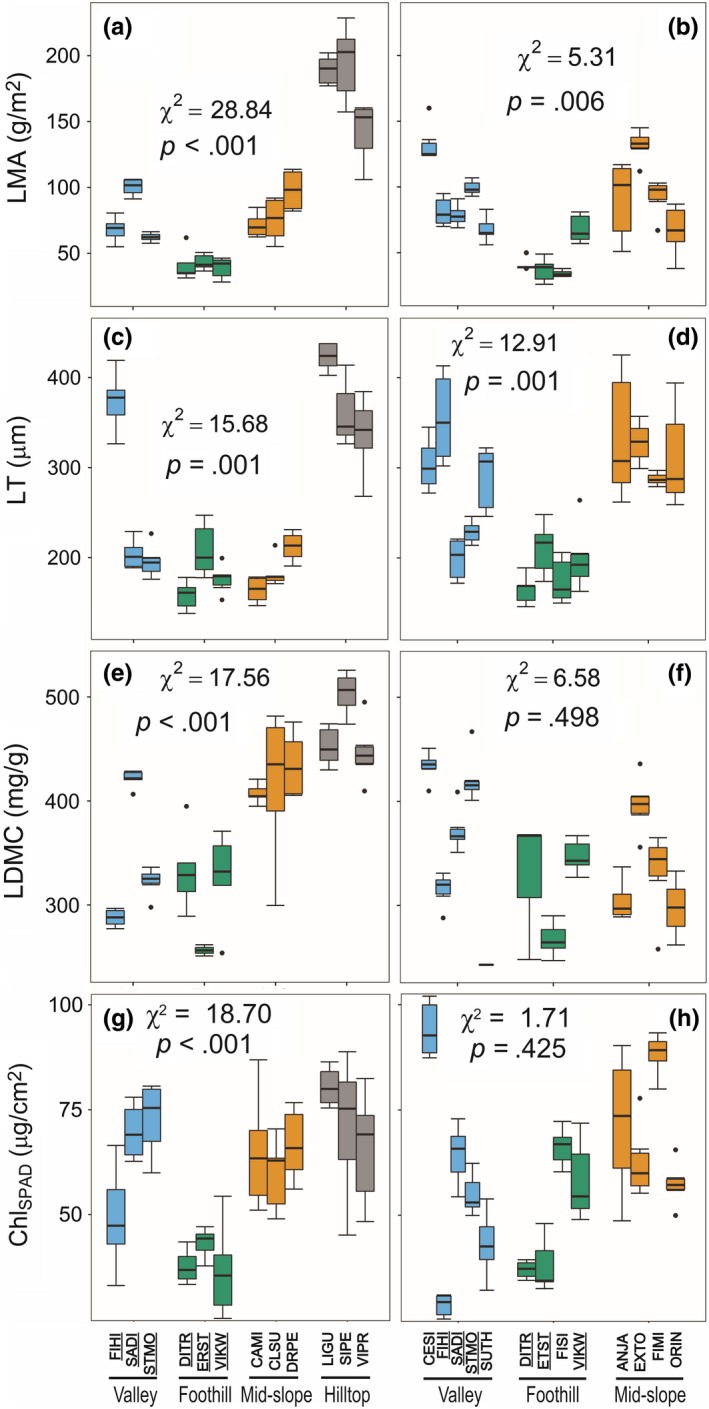
Variation in leaf traits between Nonggang National Nature Reserve (karst forest left side) and the arboretum (right side) and across habitat affinity classes (valley, foothill, mid‐slope, and hilltop). (a, b) leaf mass per area (LMA), (c, d) lamina thickness (LT), (e, f) leaf dry matter content (LDMC), (g, h) chlorophyll content estimated from SPAD meter (Chl_SPAD_). Species are indicated by four‐letter codes (explained in Table 1), and those underlined are common to both sampling sites. Chi‐square values are linear mixed effect model inferences about habitat affinity as a fixed effect

Within the relatively narrow elevation range in the karst forest (130–607 m asl), LMA differed by approximately 10‐fold (28.3–228.6 g/m^2^) among species (Figure 3a) from the lowest values found in foothill species to the highest values of hilltop species. The high LMA values of hilltop species appeared to reflect high values of both lamina thickness (Figure 3c) and LDMC (Figure 3e). Although mid‐slope species had high LDMC similar to hilltop species, their low LT values resulted in relatively low LMA values similar to valley species (Figure 3a). There were twofold (11.41–5.79 mm/mm^2^) variation in VLA, threefold (420–128 mm^−2^) variation in SD, and fivefold (.06–.33) variation in SPI (Table S2). But habitat affinity did not explain these among‐species variations.

**Figure 4:**
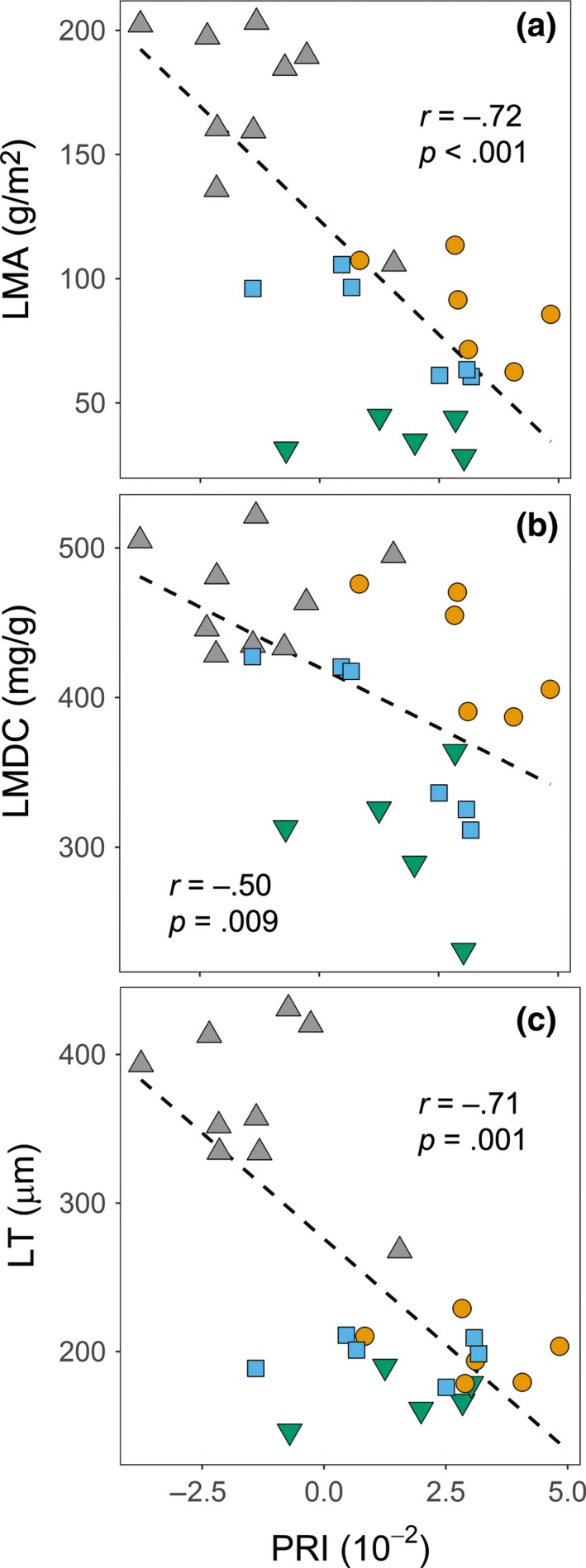
Trait–trait correlations across 36 individuals of 12 species naturally growing in Nonggang National Nature Reserve for (a) leaf mass per area versus photochemical reflectance index (PRI), (b) leaf dry matter content (LDMC) versus PRI, and (c) lamina thickness (LT) versus PRI. Habitat affinity classes are indicated by symbols: valley (square), foothill (downward triangle), mid‐slope (circle), and hilltop (upward triangle). Pearson's correlation coefficient (*r*) is shown, along with the linear regression line

Overall, optical traits that contributed to PC2 were less differentiated among habitats than LMA, LDMC, and LT that contribute to PC1. Of these Chl_SPAD_ (Figure 3g) and PRI (χ^2^ = 18.00, *p* < .001, Table S2) showed significant differences in relation to habitat affiliation. The Chl_SPAD_, which is an estimate of chlorophyll per leaf area, generally tracked the pattern of leaf thickness, except that mid‐slope species growing on shaded slopes, showed high values for their relatively thin leaves. Mid‐slope species also showed higher PRI values than those from exposed hilltops (Table S2), indicating that they exhibited greater light use efficiency. PRI showed significant negative correlations with LMA, LT, and LDMC (Figure 4a–c). Water band index (WBI) was positively correlated with LT and LMA (Table S3), indicating higher water contents per unit leaf area in thick leaves of hilltop species, despite their high LDMC.

### 3.2 Traits syndromes in the arboretum

In the arboretum, LMA tended to be the lowest for foothill species (Figure 3b), similarly to the in situ pattern in the karst forest (Figure 3a). There were no clear differences in LDMC among habitat classes of mid‐slope, foothill, and valley in the arboretum, but LT followed the pattern similar to LMA (*r *=* *.65, *p* < .001, Figure 3d). In the arboretum, PRI did not correlate with LMA (*r *=* *.05, *p* = .67), LT (*r *=* *−.21, *p* = .08) or LDMC (*r *=* *.02, *p* = .87). Among‐species variation in VLA (threefold, 3.99–12.89 mm/mm^2^), SD (sixfold, 97–585 mm^−2^), and SPI (sixfold, 0.10–0.36) did not differ significantly among habitat affinity classes (Table S2).

### 3.3 Leaf trait consistency between the sites

**Figure 5:**
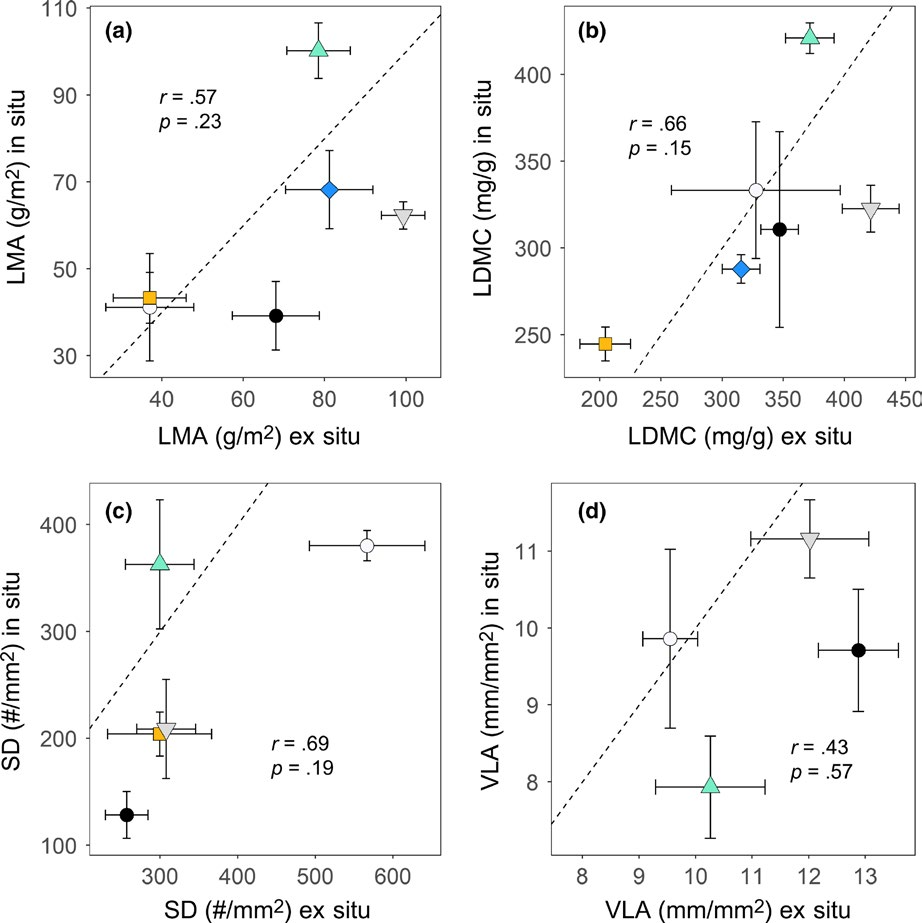
Correlations between trait values measured ex situ (the arboretum) and in situ (karst forest at Nonggang National Nature Reserve) for six tree species sampled at both sites. (a) LMA = leaf mass per area, (b) LDMC = leaf dry matter content, (c) SD = stomatal density, and (d) VLA = vein length per area. Each point indicates the species mean with standard error. Filled circle: DITR, filled square: ERST, filled diamond: FIHI, downward triangle: STMO, solid circle: VIKW, and upward triangle: SADI. Pearson's correlation coefficient (*r*) is shown. Points away from 1:1 arbitrary lines depict greater plasticity in ex situ or in situ. See Table 1 for full species names

Leaf trait values of the six species sampled in both the arboretum and karst forest (VLA, LMA, LDMC, SD, and optical traits) showed substantial differences within some species (Figure 5). These six species belonged to the valley and foothill habitats. Trait value differences within species were quantitatively assessed as the degree of departure from the 1:1 line of the mean trait values of the karst forest (in situ) plotted against those from the arboretum (ex situ). The data points for some species were close to the 1:1 line, indicating minor degrees of trait value differences, but others showed substantial departures from 1:1 line. The direction and magnitudes of such differences between the sampling sites were variable (Figure 5a–d). Further, cross‐species correlations between the sampling sites were not significant in many traits (Table 2). Exceptions were lamina thickness, SPI, and GCL, which strongly correlated between the two sampling sites (Table 2, Fig. S2), even though stomata density was higher in the arboretum in four of five species (Figure 5c).

## 4 DISCUSSION

Our comparative study of tropical karst tree species found that lamina thickness and LDMC differed significantly among habitat affinities, resulting in the highest LMA values for the hilltop species (Figure 3a). At the hilltop, trees were growing on largely soil‐less substrates and their roots were extended into the crevices in the limestone rocks. The hilltop species had not been planted in the arboretum. As we could not confirm whether species sampled in the karst forest and the arboretum were from the same parent populations, we could not distinguish whether variation in LMA in ex situ versus in situ was due to plasticity or genotypic difference (Figure 5a). Among the four habitat affinity classes, we expected that species from foothill species, where soil nutrients and moisture are the least limiting without seasonal flooding, should exhibit more acquisitive trait values. This prediction was supported by the results showing the lowest values of LMA and lamina thickness for foothill species from both the karst forest and the arboretum (Figure 3a–d). Contrary to our expectation that differences in dry season water availability should influence anatomical traits relevant for leaf hydraulics, VLA, SD, and SPI, did not show significant differences among habitats.

### 4.1 LMA as an indicator of a conservative ecological strategy

For a given leaf, LMA is a product of leaf density (dry mass per unit volume, which tightly correlates with LDMC) and lamina thickness (e.g., Kitajima & Poorter, 2010). Hence, LDMC and lamina thickness are equally important in explaining variations of LMA across a broad range of tropical tree species (e.g., Westbrook et al., 2011) as found in our study. Values of LMA generally increase with elevation (Poorter et al., 2009; Read, Moorhead, Swenson, Bailey, & Sanders, 2014). In our dataset, LMA was the lowest, not among species from the lowest elevation (the valley), but among species affiliated with the foothill, where soil volumetric water content was the highest (Fig. S1). We could not measure the nutrient availability in the rooting zone of our study species, but it was likely that soil and water availability covaried, as in sand‐ or karst‐dominated habitats in general (Cavender‐Bares, Kitajima, & Bazzaz, 2004; Mi, Li, Chen, Xie, & Bai, 2015; Zhang et al., 2007). Collectively, high LMA among hilltop species reflected overall resource‐poor conditions in hilltop habitats, suggesting LMA as an indicator of a conservative ecological strategy in karst habitats.

### 4.2 Leaf optical traits in multi‐trait syndromes

In the multivariate leaf trait correlations, optical traits measured in our study, including estimates of chlorophyll contents and xanthophyll cycle pigments, exhibited partially independent variation from LMA. Our purpose was not to measure dynamic in situ response of these traits, but to compare species under a standardized protocol. Lower PRI values indicate greater quantities of xanthophyll cycle pigments which are involved in photoprotection from excess radiation (Filella et al., 1996; Gamon et al., 1997). Low PRI values among hilltop species (Figure 4) where species were grown under fully exposed sunlight indicate high levels of photoprotection mechanisms with xanthophyll cycle pigments, low photosynthetic light use efficiency, and/or old leaf age. High LMA is a good correlate of leaf lifespan (Russo & Kitajima 2016). Hence, in our study, leaf age was likely to be more advanced for hilltop species with high LMA than foothill and valley species.

Many tropical karst hills are remotely located and difficult to access due to their rugged terrains. A database compiling optical traits and imaging technologies should be particularly useful for such ecosystems (Asner et al., 2011). Significance of negative correlations of PRI with LMA, LDMC, and LT (Figure 4a–c, *r* ^2^ ranging from .25 to .49 Table S3) suggests a possible use of PRI as a predictor of LMA. This significance owes largely to hilltop species with high LMA, LT, and low PRI (Figure 4a–c). Spectral signatures from a wider range of 400 to 2,500 nm predict specific leaf area, the inverse of LMA, with *r* ^2^ = .79 for 42 rainforest species (Asner and Martin, 2009). Prediction of LMA with optical measurements with a relatively inexpensive spectrometer (400–1,050 nm), as used in our study, may also be useful in ecological studies.

### 4.3 Leaf trait plasticity

Some of the leaf traits in our study showed substantial within‐species differences between the two study sites, which is most likely due to plasticity, but direction and magnitude of plastic responses were inconsistent across species (Figure 5). Among the leaf traits included in our study, stomatal pore index and lamina thickness exhibited low plasticity (Fig. S2), suggesting that these traits can be sampled as inherent traits, although Cordell et al. (1998) reported a wide degree of plasticity of lamina thickness in *Metrosideros polymorpha* in Hawaii. In our study, we sampled mature and fully expanded leaves from well‐exposed branches of mature trees. The main difference between the karst forest and the arboretum was that the plants in the latter experienced less degrees of drought and nutrient stress due to deeper soil and frequent watering. Possibly in response to this more favorable environment in the arboretum, four of the five species showed higher SD, and three of the four showed higher VLA than at the karst forest. Limited sample size (three species each from foothill and valley), the lack of measurements of microenvironments, leaf age, ontogeny, and population sources of plants growing in the arboretum, limit the functional implications of these results.

In conclusion, leaf traits of karst tree species exhibit syndromes that apparently evolved in relation to their specialization to edaphic habitats. In particular, specialization to hilltops, where water and nutrients may be in limited supply, is associated with a conservative ecological strategy represented by high values of LMA, lamina thickness, LDMC, and low values of chlorophyll, photochemical reflectance index. Although we also expected, vein and stomatal traits associated with leaf hydraulic properties had no significant differences among contrasting types of habitat specialization. Substantial differences in values of some leaf traits between the natural habitat and the arboretum suggest that it is not often possible to infer trait values from ex situ samples. Hence, further exploration of the relationships of optical traits with other leaf functional traits will contribute to better understanding of adaptive trait syndromes in relation to habitat specialization, especially of species that specialize in difficult to access sites, such as the karst hilltops in our study.

## CONFLICT OF INTEREST

None declared.

## DATA ACCESSIBILITY

Data presented in this manuscript is available via Dryad repository (https://doi.org/10.5061/dryad.ft55t).

## AUTHOR CONTRIBUTIONS

NG, KK, and UMG conceived of the ideas and designed methods; NG collected the data; all authors contributed to data analysis and manuscript writing.
