# Supplementary figures and images for: Leaf trait variations associated with habitat affinity of tropical karst tree species
Source: Ecol Evol. 2017 Nov 28;8(1):286–95. doi: 10.1002/ece3.3611 (PMC5756878; doi:10.1002/ece3.3611)

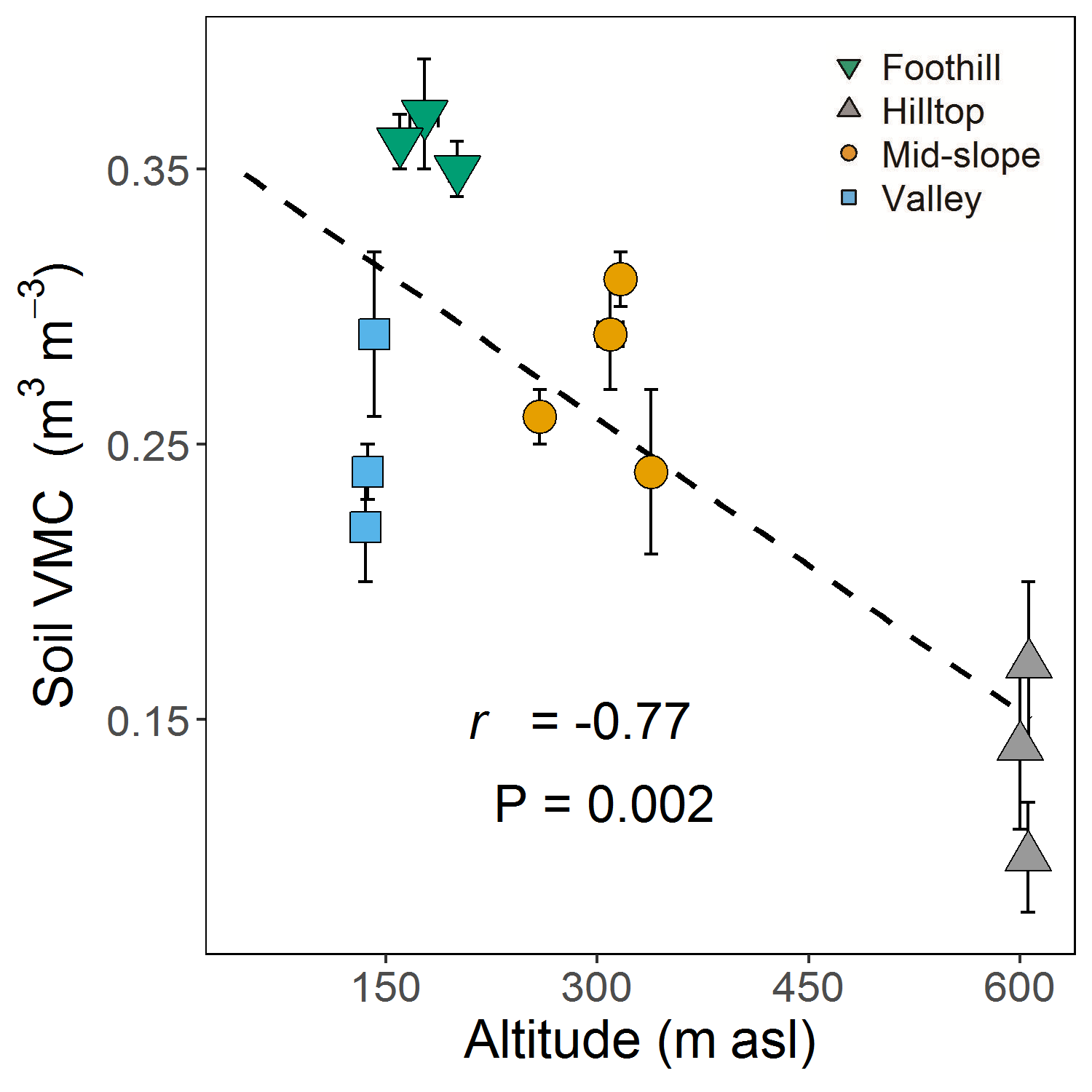

Supplement: Supplementary file 1 [file ECE3-8-286-s001.tiff]

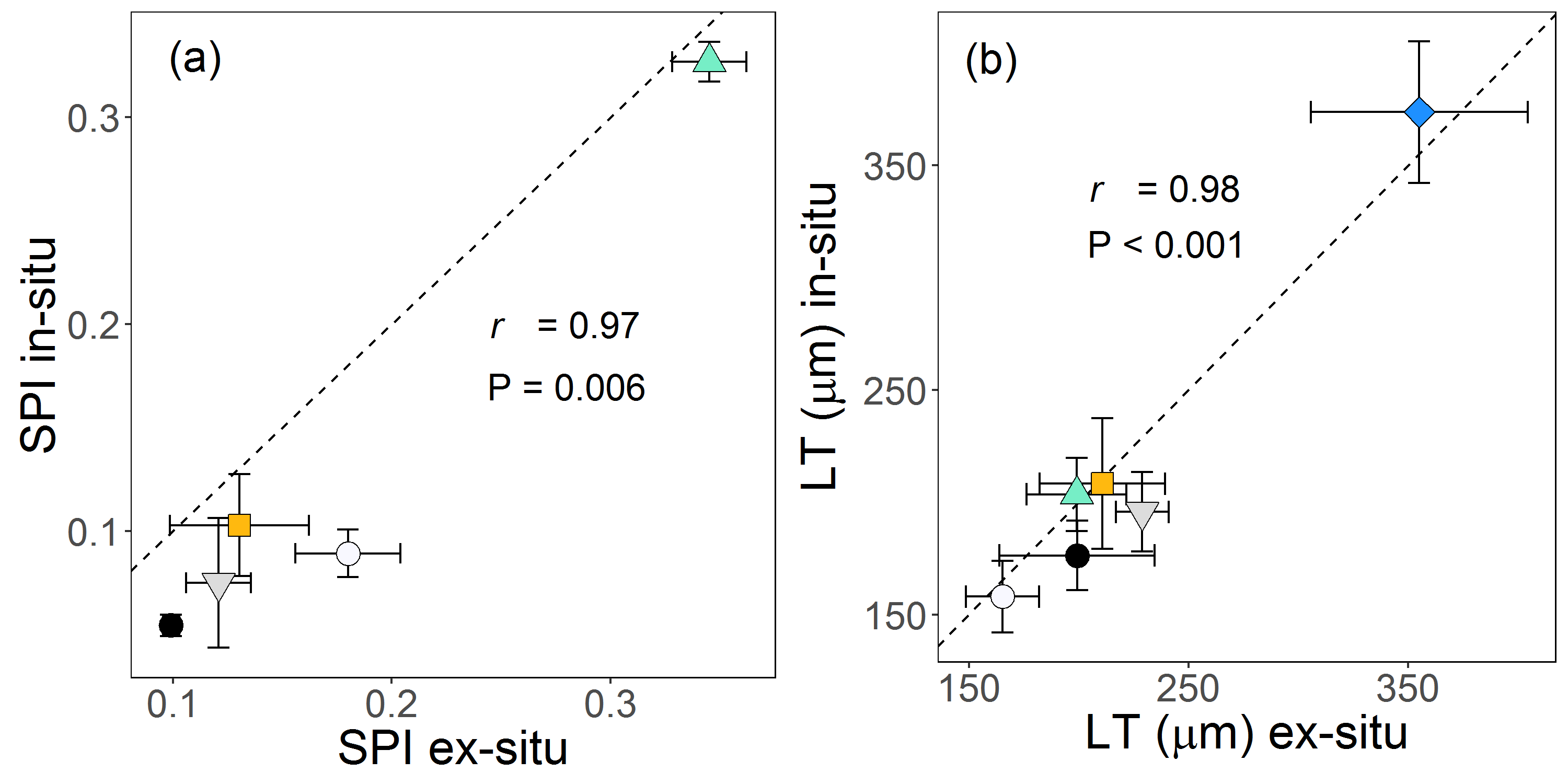

Supplement: Supplementary file 2 [file ECE3-8-286-s002.tiff]
