# Supplementary material for: Leaf trait variations associated with habitat affinity of tropical karst tree species
Source: Ecol Evol. 2017 Nov 28;8(1):286–95. doi: 10.1002/ece3.3611 (PMC5756878; doi:10.1002/ece3.3611)
Supplement: Supplementary file 3 [file ECE3-8-286-s003.docx]

**TABLE S1.** Climatological and other environmental parameters of the arboretum at Qing Xiu Shan and the karst forest at Nonggang National Nature Reserve, Guangxi, Southwestern China

| Parameter | Karst forest (*in-situ*) | Arboretum (*ex-situ*) |
| --- | --- | --- |
| Average annual rainfall range (mm/year) | 1200-1500 | ~1400 |
| Average ambient temperature (°C) | 22 | 21 |
| Relative humidity (%) | 92-94 | - |
| Dry season | October-February | October-February |
| Area (ha) | 10,080 | 1,354 |
| GPS location | 22°28' N 106°57' E | 22°46' N 108°33' E |
| Average elevation above mean sea level (m) | 118-680 | 80-289 |
| Topography | deep karsts, peak cluster depressions and valleys | rolling to undulating |

**TABLE S2.** Means ± standard errors of 13 ecophysiological traits measured *in-situ* in Nonggang National Nature Reserve (karst forest) and *ex-situ* in Qing Xiu Shan, Nanning (arboretum) in 19 karst tree species

| Habitat | Species | LA (cm^2^) | | SD (# / mm^2^) | | GCL (µm) | | SPI (unitless) | | VLA (mm mm^−2^) | |
| --- | --- | --- | --- | --- | --- | --- | --- | --- | --- | --- | --- |
|  |  | Arboretum | Karst forest | Arboretum | Karst forest | Arboretum | Karst forest | Arboretum | Karst forest | Arboretum | Karst forest |
| Valley | CESI | 35.91±5.43 | NA | 464.7±50.8 | NA | 22.45±0.43 | NA | 0.24±0.03 | NA | 5.83±0.20 | NA |
|  | FIHI | 117.08±8.88 | 140.57±26.58 | NA | NA | NA | NA | NA | NA | NA | NA |
|  | SADI | 128.29±14.97 | 184.09±24.78 | 299.7±18.0 | 362.5±24.7 | 34.41±1.59 | 30.35±0.80 | 0.36±0.03 | 0.33±0.01 | 10.26±0.40 | 7.94±0.28 |
|  | STMO | 164.13±24.05 | 143.84±26.02 | 307.8±15.5 | 208.6±20.7 | 21.18±1.45 | 19.83±0.58 | 0.14±0.02 | 0.08±0.02 | 12.03±0.43 | 11.16±0.21 |
|  | SUTH | 151.81±20.26 | NA | NA | NA | NA | NA | NA | NA | 3.99±0.12 | NA |
| Foothill | DITR | 110.92±19.45 | 203.42±9.16 | 585.2±39.9 | 380.0±5.8 | 17.62±0.50 | 15.65±0.61 | 0.18±0.01 | 0.10±0.01 | 9.56±0.20 | 9.86±0.48 |
|  | ERST | 92.27±15.54 | 148.95±10.41 | 299.0±27.4 | 203.9±8.4 | 20.85±0.54 | 22.34±1.06 | 0.14±0.02 | 0.11±0.02 | 8.91±0.48 | NA |
|  | FISI | 160.50±11.14 | NA | NA | NA | NA | NA | NA | NA | 7.57±0.37 | NA |
|  | VIKW | 17.25±2.07 | 15.85±1.97 | 257.0±11.2 | 128.3±9.7 | 20.47±0.62 | 20.75±0.88 | 0.11±0.02 | 0.06±0.01 | 12.89±0.29 | 9.72±0.33 |
| Mid-slope | ANJA | 37.37±5.92 | NA | 97.0±11.2 | NA | 31.93±1.41 | NA | 0.10±0.02 | NA | 5.32±0.23 | NA |
|  | CAMI | NA | 15.58±1.7 | NA | NA | NA | NA | NA | NA | NA | NA |
|  | CLSU | NA | 26.12±1.88 | NA | 239.8±17.7 | NA | 25.18±1.47 | NA | 0.16±0.02 | NA | 11.41±0.29 |
|  | DRPE | NA | 20.82±1.52 | NA | 419.9±25.7 | NA | 21.77±0.68 | NA | 0.18±0.03 | NA | 8.83±0.18 |
|  | EXTO | 113.53±14.03 | NA | 394.7±32.4 | NA | 24.67±1.24 | NA | 0.25±0.03 | NA | 8.81±0.60 | NA |
|  | FIMI | 19.62±0.71 | NA | 178.2±14.6 | NA | 29.99±2.27 | NA | 0.17±0.04 | NA | 7.38±0.08 | NA |
|  | ORIN | 64.06±6.11 | NA | 488.3±34.6 | NA | 21.94±0.67 | NA | 0.24±0.02 | NA | 8.17±0.24 | NA |
| Hilltop | LIGU | NA | 16.22±1.35 | NA | 397.5±15.4 | NA | 22.14±1.04 | NA | 0.18±0.02 | NA | 5.79±0.43 |
|  | SIPE | NA | 16.55±1.42 | NA | 184.6±20.2 | NA | 21.13±0.45 | NA | 0.09±0.01 | NA | 10.09±0.25 |
|  | VIPR | NA | 14.24±1.76 | NA | 288.2±9.8 | NA | 29.43±0.99 | NA | 0.27±0.03 | NA | 6.29±0.27 |

**TABLE S2.** Continued from previous page

| Habitat | Species | Chl_SPAD_ (µg cm^−2^) | | LMA (g m^−2^) | | LT (µm) | | LDMC (mg g^−1^) | |
| --- | --- | --- | --- | --- | --- | --- | --- | --- | --- |
|  |  | Arboretum | Karst forest | Arboretum | Karst forest | Arboretum | Karst forest | Arboretum | Karst forest |
| Valley | CESI | 94.06±2.69 | NA | 132.34±5.85 | NA | 303.50±11.61 | NA | 433.84±5.56 | NA |
|  | FIHI | 28.64±1.06 | 49.11±4.84 | 81.17±4.39 | 68.17±3.68 | 355.00±20.17 | 373.56±12.87 | 315.50±6.30 | 287.72±3.36 |
|  | SADI | 64.51±2.81 | 69.75±2.66 | 78.50±3.19 | 100.18±2.86 | 199.00±9.29 | 203.45±6.68 | 371.84±8.10 | 420.94±3.95 |
|  | STMO | 54.89±1.96 | 72.94±3.49 | 99.34±2.19 | 62.29±1.29 | 228.84±4.89 | 195.67±7.21 | 421.50±9.49 | 322.53±5.48 |
|  | SUTH | 43.10±3.11 | NA | 68.00±4.53 | NA | 289.40±15.94 | NA | 214.20±9.02 | NA |
| Foothill | DITR | 37.11±0.83 | 37.67±1.84 | 37.00±4.41 | 41.12±5.52 | 165.20±7.46 | 158±7.15 | 327.67±39.84 | 333.22±17.64 |
|  | ERST | 37.94±2.62 | 43.30±1.41 | 37.00±3.65 | 43.29±2.41 | 210.67±11.66 | 208.28±11.90 | 204.50±8.46 | 244.56±4.00 |
|  | FISI | 66.22±1.79 | NA | 34.17±0.99 | NA | 174.00±10.11 | NA | 267.34±6.34 | NA |
|  | VIKW | 58.05±3.82 | 36.44±4.42 | 68.00±4.37 | 39.19±3.23 | 199.17±14.41 | 176.33±6.34 | 347.00±6.29 | 310.57±23.01 |
| Mid-slope | ANJA | 71.89±6.72 | NA | 90.67±12.03 | NA | 333.50±29.39 | NA | 274.34±20.78 | NA |
|  | CAMI | NA | 64.79±5.45 | NA | 71.27±3.61 | NA | 164.28±5.90 | NA | 407.19±4.37 |
|  | CLSU | NA | 59.66±3.91 | NA | 75.74±6.68 | NA | 182.44±6.39 | NA | 418.22±28.78 |
|  | DRPE | NA | 66.68±3.44 | NA | 97.99±6.19 | NA | 212.22±6.48 | NA | 434.57±12.69 |
|  | EXTO | 62.55±3.42 | NA | 131.84±4.59 | NA | 328.17±9.13 | NA | 396.50±10.66 | NA |
|  | FIMI | 88.23±1.96 | NA | 92.67±5.55 | NA | 287.50±2.71 | NA | 332.34±15.96 | NA |
|  | ORIN | 57.45±2.06 | NA | 67.00±7.61 | NA | 310.67±22.95 | NA | 253.67±24.61 | NA |
| Hilltop | LIGU | NA | 80.48±1.89 | NA | 189.24±4.90 | NA | 422.93±6.93 | NA | 452.24±7.55 |
|  | SIPE | NA | 71.22±6.60 | NA | 195.12±11.66 | NA | 359.72±14.43 | NA | 503.68±8.10 |
|  | VIPR | NA | 65.88±5.46 | NA | 142.69±9.28 | NA | 336.84±16.94 | NA | 446.73±11.49 |

**TABLE S2.** Continued from previous page

| Habitat | Species | NDVI (unitless) | | WBI (unitless) | | MCARI (unitless) | | PRI (×10^-2^) | |
| --- | --- | --- | --- | --- | --- | --- | --- | --- | --- |
|  |  | Arboretum | Karst forest | Arboretum | Karst forest | Arboretum | Karst forest | Arboretum | Karst forest |
| Valley | CESI | 0.84±0.01 | NA | 0.87±0.01 | NA | 7.41±0.81 | NA | 3.65±0.41 | NA |
|  | FIHI | 0.76±0.01 | 0.84±0.02 | 0.86±0.01 | 1.03±0.01 | 27.72±1.53 | 20.47±2.67 | −0.62±0.08 | 0.81±0.00 |
|  | SADI | 0.82±0.02 | 0.86±0.01 | 0.90±0.02 | 1.05±0.01 | 8.51±1.53 | 15.62±1.19 | 4.04±0.18 | 0.80±0.01 |
|  | STMO | 0.77±0.02 | 0.84±0.01 | 0.91±0.03 | 1.03±0.01 | 13.46±1.01 | 7.20±1.30 | 0.49±0.02 | 2.86±0.24 |
|  | SUTH | 0.79±0.01 | NA | 0.88±0.01 | NA | 20.48±3.74 | NA | 1.52±0.33 | NA |
| Foothill | DITR | 0.77±0.01 | 0.78±0.02 | 0.86±0.01 | 1.01±0.01 | 11.69±0.89 | 25.54±5.65 | 0.95±0.13 | −0.46±0.01 |
|  | ERST | NA | 0.77±0.03 | NA | 1.06±0.01 | NA | 10.23±1.79 | NA | 0.74±0.01 |
|  | FISI | 0.78±0.01 | NA | 0.85±0.01 | NA | 4.41±0.41 | NA | 3.55±0.07 | NA |
|  | VIKW | 0.82±0.01 | 0.80±0.02 | 0.87±0.01 | 1.01±0.01 | 6.76±0.98 | 16.56±3.70 | 3.43±0.21 | 2.14±0.42 |
| Mid-slope | ANJA | 0.87±0.01 | NA | 0.95±0.03 | NA | 7.96±0.86 | NA | 4.41±0.37 | NA |
|  | CAMI | NA | 0.84±0.02 | NA | 1.01±0.01 | NA | 6.66±1.08 | NA | 3.24±0.25 |
|  | CLSU | NA | 0.84±0.02 | NA | 1.01±0.01 | NA | 6.66±1.08 | NA | 3.24±0.25 |
|  | DRPE | NA | 0.86±0.01 | NA | 1.01±0.01 | NA | 10.19±2.45 | NA | 3.19±0.63 |
|  | EXTO | 0.87±0.01 | NA | 0.93±0.02 | NA | 8.95±0.88 | NA | 1.85±0.50 | NA |
|  | FIMI | 0.85±0.01 | NA | 0.89±0.01 | NA | 9.66±0.64 | NA | 3.40±0.33 | NA |
|  | ORIN | 0.8±0.02 | NA | 0.88±0.01 | NA | 11.02±1.14 | NA | 1.10±0.38 | NA |
| Hilltop | LIGU | NA | 0.81±0.02 | NA | 1.06±0.02 | NA | 10.07±1.77 | NA | −0.56±0.00 |
|  | SIPE | NA | 0.81±0.02 | NA | 1.04±0.01 | NA | 14.39±2.55 | NA | −2.54±0.01 |
|  | VIPR | NA | 0.84±0.03 | NA | 1.07±0.02 | NA | 9.58±2.56 | NA | −0.58±0.00 |

**TABLE S3.** Trait-trait correlation between 13 leaf ecophysiological traits of 36 individuals of 12 dominant karst tree species in Nonggang National Nature Reserve. Values in the upper diagonal are Pearson’s product-moment correlation coefficients (*r*) and their *P* values are in the lower diagonal, with bold letters indicating significant correlations (*P*<0.05).

|  | LA | LMA | LDMC | LT | PRI | MCARI | NDVI | WBI | Chl_SPAD_ | SD | GCL | SPI | VLA |
| --- | --- | --- | --- | --- | --- | --- | --- | --- | --- | --- | --- | --- | --- |
| LA |  | **−0.43** | **−0.40** | **−0.50** | 0.06 | 0.29 | 0.07 | 0.08 | −0.06 | 0.14 | −0.06 | 0.11 | 0.20 |
| LMA | **0.027** |  | **0.81** | **0.93** | **−0.72** | −0.11 | −0.23 | **0.52** | **0.65** | 0.26 | 0.23 | 0.31 | **−0.58** |
| LDMC | **0.042** | **<0.001** |  | **0.61** | **−0.50** | −0.24 | 0.03 | 0.29 | **0.64** | 0.30 | **0.41** | **0.46** | **−0.47** |
| LT | **0.009** | **<0.001** | **0.001** |  | **−0.71** | −0.08 | −0.31 | **0.54** | **0.53** | 0.18 | 0.13 | 0.18 | **−0.64** |
| PRI | 0.788 | **<0.001** | **0.009** | **<0.001** |  | **−0.44** | **0.49** | −**0.39** | −0.26 | −0.11 | −0.22 | −0.26 | **0.48** |
| MCARI | 0.151 | 0.577 | 0.235 | 0.713 | **0.024** |  | **−0.43** | −0.19 | **−0.44** | 0.03 | −0.13 | −0.06 | 0.06 |
| NDVI | 0.737 | 0.269 | 0.883 | 0.125 | **0.011** | **0.030** |  | −0.11 | 0.20 | 0.14 | **0.41** | **0.39** | −0.02 |
| WBI | 0.694 | **0.007** | 0.144 | **0.004** | **0.047** | 0.341 | 0.583 |  | **0.42** | 0.09 | 0.19 | 0.20 | **−0.42** |
| Chl_SPAD_ | 0.768 | **<0.001** | **<0.001** | **0.005** | 0.201 | **0.025** | 0.325 | **0.033** |  | 0.32 | 0.23 | **0.39** | **−0.44** |
| SD | 0.496 | 0.203 | 0.139 | 0.370 | 0.583 | 0.888 | 0.491 | 0.648 | 0.108 |  | 0.08 | **0.63** | **−0.48** |
| GCL | 0.755 | 0.253 | **0.038** | 0.515 | 0.275 | 0.523 | **0.038** | 0.364 | 0.257 | 0.697 |  | **0.81** | **−0.48** |
| SPI | 0.600 | 0.123 | **0.017** | 0.367 | 0.206 | 0.767 | **0.051** | 0.325 | **0.048** | **0.001** | **<0.001** |  | **−0.66** |
| VLA | 0.316 | **0.002** | **0.015** | **<0.001** | **0.012** | 0.754 | 0.915 | **0.034** | **0.026** | **0.013** | **0.013** | **<0.001** |  |
