# Supplementary material for: Leaf trait variations associated with habitat affinity of tropical karst tree species
Source: Ecol Evol. 2017 Nov 28;8(1):286–95. doi: 10.1002/ece3.3611 (PMC5756878; doi:10.1002/ece3.3611)
Supplement: Supplementary file 4 [file ECE3-8-286-s004.docx]

**APPENDIX S1.** Equations for calculating optical traits

We used the SpectraSnap v1.1.3.150 software (CID Bioscience, Camas, Washington, USA) connected to a leaf spectrometer (CID Bioscience, Camas, Washington, USA) to record light reflectance, transmission and absorption spectra covering visible (400-700 nm) to near infra-red (700-950 nm) wavelengths at 0.21 nm optical resolution. To reduce data noise, we obtained mean of five spectral readings, by setting integration time to 300 ms and Boxcar width to 10 pixels, and averaging adjacent pixels for smoothing. Then Photochemical Reflectance Index (PRI: Gamon, Peñuelas and Field, 1992), Modified Chlorophyll Absorption Ratio Index (MCARI: Daughtry, Walthall and Kim, 2000), Normalized Difference Vegetation Index (NDVI: Rouse *et al.*, 1974) and Water Band Index (WBI: Peñuelas *et al.*, 1993) were calculated from recorded spectra using following equations (1-4). The lowercase rho (*ρ*) and subscripted number within equations indicate wavelengths in nanometres. The SPAD readings (SPAD-502 Konica Minolta, Osaka, Japan) were converted to area based chlorophyll contents using equation 5 (Coste *et al.*, 2010).

$PRI = \frac{\rho_{531}-\rho_{570}}{\rho_{531}+\rho_{570}}$ Equation 01

$MCARI = \left[ \left( \rho_{700}-\rho_{670} \right)-0.2\left( \rho_{700}-\rho_{550} \right) \right]\times\left( \frac{\rho_{700}}{\rho_{670}} \right)$ Equation 02

$NDVI=\frac{\left( NIR-Red \right)}{\left( NIR+Red \right)}$ Equation 03

$WBI = \frac{\rho_{970}}{\rho_{900}}$ Equation 04

${Chl}_{{SPAD}_{i}}= \frac{117.1 \times{SPAD}_{i}}{148.84- {SPAD}_{i}}$ Equation 05

**REFERENCES FOR APPENDIX S1**

Coste, S. *et al.* (2010) ‘Assessing foliar chlorophyll contents with the SPAD-502 chlorophyll meter: a calibration test with thirteen tree species of tropical rainforest in French Guiana’, *Annals of Forest Science*, 67(6), pp. 607–607.

Daughtry, C. S. T., Walthall, C. L. and Kim, M. S. (2000) ‘Estimating corn leaf chlorophyll concentration from leaf and canopy reflectance’, *Remote Sensing of environment*, 74(2), pp. 229–239.

Gamon, J. A., Peñuelas, J. and Field, C. B. (1992) ‘A narrow-waveband spectral index that tracks diurnal changes in photosynthetic efficiency’, *Remote Sensing of Environment*, 41(1), pp. 35–44.

Peñuelas, J. *et al.* (1993) ‘The reflectance at the 950–970 nm region as an indicator of plant water status’, *International Journal of Remote Sensing*, 14(10), pp. 1887–1905.

Rouse, J. W. *et al.* (1974) ‘Monitoring vegetation systems in the Great Plains with Third Earth Resources Technology Satellite’, 351, p. 309.
